# Stochastically evolving graphs via edit semigroups

**DOI:** 10.1073/pnas.2526595122

**Published:** 2025-11-26

**Authors:** Fan Chung, Sawyer Jack Robertson

**Affiliations:** ^a^Department of Mathematics, University of California San Diego, La Jolla, CA 92093

**Keywords:** Markov chains, mixing times, left regular bands, spectral graph theory, random graphs

## Abstract

We investigate a randomly evolving process of subgraphs in an underlying host graph using the spectral theory of semigroups related to the Tsetlin library and hyperplane arrangements. Starting with some initial subgraph, at each iteration, we apply a randomly selected edit to the current subgraph. Such edits vary in nature from simple edits consisting of adding or deleting an edge, or compound edits which can affect several edges at once. This evolving process generates a random walk on the set of all possible subgraphs of the host graph. We show that the eigenvalues of this random walk can be naturally indexed by subsets of edges of the host graph. We also provide, in the case of simple edits, a closed-form formula for the eigenvectors of the transition probability matrix and a sharp bound for the rate of convergence of this random walk. We consider extensions to the case of compound edits; examples of this model include the previously studied Moran forest model and a dynamic random intersection graph model. Evolving graphs arise in a variety of fields ranging from deep learning and graph neural networks to epidemic modeling and social networks. Our random evolving process serves as a general stochastic model for sampling random subgraphs from a given graph.

## Significance

Many real-world networks change dynamically but can be notoriously difficult to study. We introduce a graph edit process that, at each step, randomly selects and applies an edit (e.g., adding or deleting an edge, or several edges in tandem) to the current subgraph within a fixed host graph. Despite the wide generality of our model, by using algebraic tools from semigroup theory, we obtain an exact spectral description of this process: closed-form eigenvalues (indexed by edge sets), closed-form eigenvectors (in the case of “simple” edits), and bounds on the mixing time of the process. These results give provable guarantees for sampling and inference on dynamic networks.

## 1. Introduction

Many problems arising in dealing with large data and deep learning involve graphs that are dynamically evolving, with edges appearing and disappearing over time. Evolving graphs can be regarded as stochastic processes that have been extensively studied in the literature with a wide range of applications (see, e.g., refs. 1 and 2). In this paper, we investigate a general edit-based model of stochastically evolving subgraphs of a specified host graph. At each step of the process, an edit is randomly chosen and applied to the current set of edges.

We consider two types of edits. A simple edit consists of adding or deleting a chosen edge, while a compound edit involves adding or deleting multiple edges at once. Our graph evolving process satisfies the following memoryless property: Namely that each chosen edit is carried out regardless of the previous edits or existing graph. For example, a simple edit such as “add edge *e*” is applied regardless of whether *e* belongs to the current subgraph, and has no effect if *e* is already present. By treating edits and their products as elements of a semigroup, we will demonstrate that the edit semigroup forms a so-called left regular band. Namely, for any two elements *x*, *y* belonging to the edit semigroup, we have $x^{2}=x$ and the following memorylessness identity holds:

$$\begin{array}{c} xyx=xy. \end{array}$$

We then apply methods from semigroup spectral theory to the evolving graph process and derive closed-form expressions for the eigenvalues, stationary distribution, and eigenvectors of the transition probability matrix, and sharp mixing time bounds.

We remark that different choices of edits give rise to distinct graph evolving processes, which in turn have their own unique stationary distributions. In particular, we show that the stationary distribution of a certain graph edit Markov chain obtained from simple edits is equal in distribution to a corresponding edge-independent random graph model, including such examples as Erdős-Rényi graphs, power law graphs, and stochastic block models (see, e.g., *Examples* 2.2 to 2.3). When the Markov chain is obtained from compound edits, the stationary distribution is more complex.

Markov chains that are carried out on state spaces such as ours consisting of subsets [see also models of vertex colorings (3, 4), or spin configurations (5, 6)], and that permit compound updates (i.e., not just nearest-neighbor flips), are famously difficult to study because the resulting dependencies often lead to transition probability matrices lacking clear symmetry or easily discernible locality properties. Nevertheless, with additional structure, one can often say quite a bit. In this case, the fact that the moves from one subgraph to another are generated by elements of a left regular band drastically streamlines the spectral analysis. It is encouraging that this is the case despite the apparent broad generality of the compound edit process framework. We consider two examples of our Markov chain obtained from applying compound edits: The first example has a stationary distribution known as the Moran forest model, which is a random forest model that has applications in computational biology (7). The second has a stationary distribution which corresponds to the random intersection graph model investigated by Godehardt and Jaworski (8). By using our semigroup-based methods, we are able to give sharp bounds on the spectral gap and the rate of convergence for both processes, which have not been done previously.

**Fig. 1.**
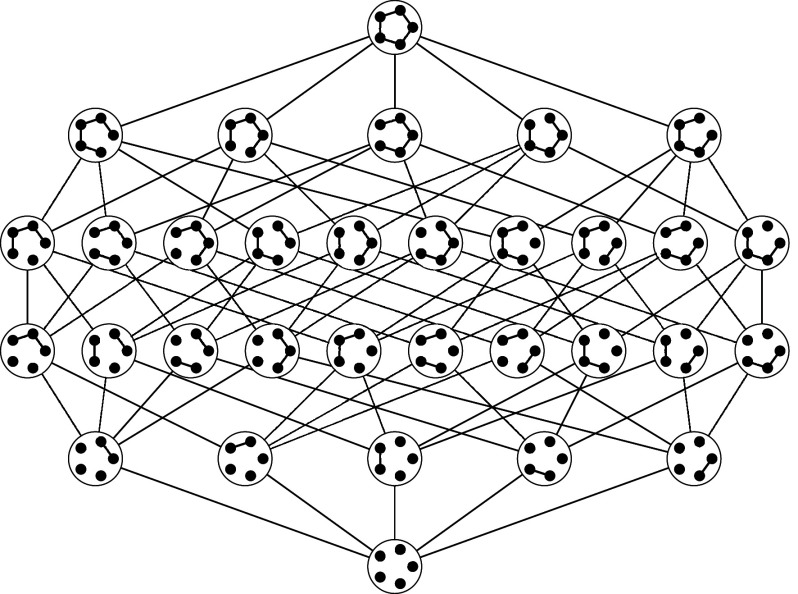
This state graph is associated with the simple edit process with the host graph corresponding to a cycle on 5 edges. Edges are directed with possibly different weights; self-loops are not shown.

**Fig. 2.**
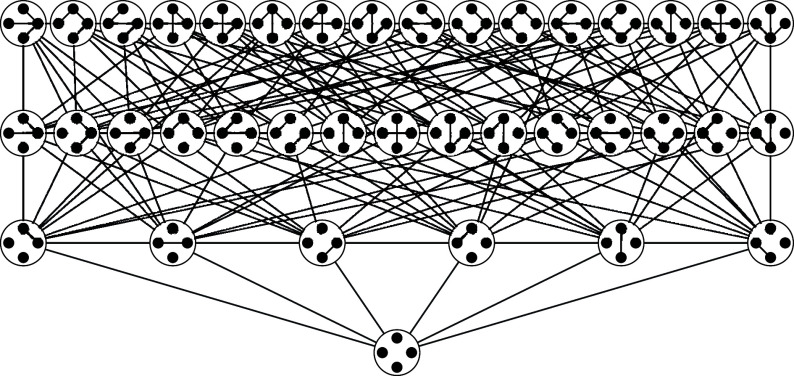
This state graph is associated with the Moran forest process with the host graph $H=K_{4}$. Edges are directed with possibly different weights; self-loops are not shown.

Our graph evolving sequence can also be regarded as a random walk on an associated directed state graph. Each node of the state graph corresponds to a subgraph of the host graph and the links of the state graph are defined and weighted by the graph evolving process of adding and deleting edges in the host graph. Two examples of state graphs formed by applying simple and compound edits are illustrated in Figs. 1 and 2, respectively. In spite of the enormous size of the state graph (up to $2^{m}$ nodes where *m* denotes the number of edges in the host graph), our methods facilitate a detailed investigation of the spectral properties of the state graph and the corresponding transition probability matrix.

Spectral analysis of random walks on semigroups dates back to Bidigare, Hanlon, and Rockmore (9), who derived a closed-form expression for the eigenvalues of the transition probability matrix of a Markov chain based on book shuffling (Tsetlin library). Brown and Diaconis later obtained convergence bounds for random walks on hyperplane arrangements (10). Brown gave a detailed treatment by exploring connections between several combinatorial structures and the eigenvalues of associated random walks (11). Saliola later analyzed eigenvectors of transition probability matrices for these walks (12). The first author and Graham used these methods for edge flipping games and voter models (13) [see also the further work (14–16)].

Stochastically evolving graph models have been studied in a variety of settings; examples include edge-switching Markov chains for sampling degree-regular graphs (2), up–down walks on simplicial complexes for sampling spanning trees and matroid bases (1), or edge-Markovian dynamic graphs (17, 18). Various temporal graph models have appeared in a variety of applied settings, including large person-to-person communication networks (19), interaction networks of molecular systems in biology (20), disease and epidemic models (21), and phylogenetic trees (7, 22), among others (23–25). Moreover, graph neural network models have been extensively studied to predict future behavior in these systems (26), with applications in areas such as automatic robot design (27), time series forecasting (28), and object detection (29), among others (30).

Before we state several main results, we need some definitions. We consider a fixed host graph H=(V,E) with a finite vertex set V and edge set $E\subseteq \left(\begin{array}{c} V\\ 2 \end{array}\right)$, and set |V|=n and |E|=m. A graph edit is an idempotent map $x:2^{E}\to 2^{E}$ (i.e., $x^{2}=x$). A *simple edit* adds or deletes a single edge.

Definition 1.1 (Simple edit process): Given $0<p_{e}<1$ for each e∈E, the simple edit process with edge probabilities ${\left(p_{e}\right)}_{e\in E}$ is defined as follows. Starting from $G_{0}=\left(V,E_{0}\right)$, for each *t* ≥ 1 choose e∈E uniformly at random and set

$$\begin{array}{c} E_{t}=\left\{\begin{array}{cc} E_{t-1}\cup \left\{e\right\}, & \text{with probability}\;p_{e},\\ E_{t-1}\setminus \left\{e\right\}, & \text{with probability}\;1\;-\;p_{e}. \end{array}\right. \end{array}$$

with $G_{t}=\left(V,E_{t}\right)$ for *t* ≥ 0.

**Fig. 3.**
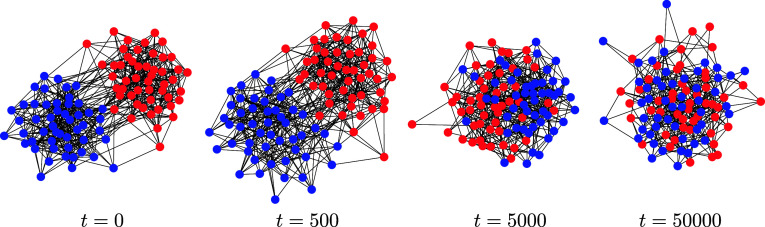
A simple graph edit process simulation on *n* = 100 nodes and host graph $H=K_{100}$ with edge probabilities $p_{e}=0.075$ for each e∈E. The initial state originates from a two-community stochastic block model with nodes colored red and blue accordingly (*Examples* 2.2 and 2.4).

A simulation of a simple edit process appears in Fig. 3.

Theorem 1.2 (Eigenvalues for the simple edit process). *Consider the simple edit process* ${\left(G_{t}\right)}_{t\geq 0}$ *defined on a host graph* H=(V,E) *with edge probabilities* ${\left(p_{e}\right)}_{e\in E}$. *Then the transition probability matrix* P *for the associated Markov chain is diagonalizable and has eigenvalues* *λ*_*T*_ *indexed by subsets* T⊆E*,* *each occurring with multiplicity one, and which have the following form:*

$$\begin{array}{c} \lambda_{T}=\frac{\left|T\right|}{\left|E\right|}, \end{array}$$

*where* |E| *is the cardinality of* E*,* *and similarly for* *T.* *Equivalently,* P *has an eigenvalue* $\frac{k}{\left|E\right|}$ *for each* 0≤k≤|E| *with multiplicity* |E|k.

*Theorem* 1.2 can be regarded as an extension of Ehrenfest’s urn model (see ref. 10, Ex. 3B.1) with additional graph structures and edge probabilities both of which are essential for the graph evolving process with compound edits later. We provide the proof *Theorem* 1.2 in Section 4.

Theorem 1.3 (Mixing time for the simple edit process). *Assume the hypotheses of *Theorem* 1.2. Let *m* denote the number of edges of* H *and let* *π* *denote the stationary distribution of the Markov chain* ${\left(G_{t}\right)}_{t\geq 0}$*.* *Then, the total variation distance of the transition probability matrix* P *satisfies*

[1] $$\begin{array}{c} \|P^{t}\left(E_{0},\cdot \right){-\;\pi \|}_{\mathrm{TV}}\leq e^{-c} \end{array}$$

*provided* t≥m(c+2logm).

*Theorem* 1.3 is proved in Section 4. We note that the bound in Eq. **1** is stronger than some well-known spectral mixing time estimates for irreducible and reversible Markov chains (see, e.g., ref. 31), which often depend on the stationary distribution *π*.

We also obtain analogous results for compound edit processes, defined below. If *x* is any graph edit, its *support* supp (x)⊆E is the subset of edges in the host graph which are edited by *x* (*Remark* 3.5).

Definition 1.4 (Compound edit process): Suppose A is a given set of graph edits and $w\in R^{A}$ is a probability distribution on A. Starting with an initial subgraph $G_{0}=\left(V,E_{0}\right)$, for each *t* ≥ 1, sample an edit x∈A according to the distribution *w* and apply *x* to the graph $G_{t-1}=\left(V,E_{t-1}\right)$. The resulting sequence of graphs ${\left(G_{t}\right)}_{t\geq 0}$ is said to be a *compound edit process obtained from* A *with distribution* *w.*

Theorem 1.5 (Eigenvalues for compound edit processes). *Let* H=(V,E) *be a fixed host graph, let* A *be a given set of edits, and let* $w\in R^{A}$ *be a given probability distribution on* A*.* *Let* P *denote the transition probability matrix of the compound edit process obtained from* A *with distribution* *w. Then* P *is diagonalizable and has eigenvalues indexed by those subsets* X⊆E *which are formed as unions of sets from* ${\left\{\mathrm{supp}\;\left(x\right)\right\}}_{x\in A}$*,* *together with the empty set, with corresponding eigenvalue*

$$\begin{array}{c} \lambda_{X}=\sum_{\begin{array}{c} x\in A\\ \mathrm{supp}\;(x)\subseteq X \end{array}}w_{x}. \end{array}$$

*The multiplicity of* *λ*_*X*_ *depends on the choice of* A *and can be computed from the Möbius function of the join semilattice generated by* ${\left\{\mathrm{supp}\;\left(x\right)\right\}}_{x\in A}$ *and set union (see refs.* *11**,* *theorem 1 and* *32**,* *section 3.7).*

*Theorem* 1.5 is proved in Section 5, and leads to an estimate on the mixing time of a compound edit process as follows.

Theorem 1.6. (Mixing time for compound edit processes). *Assume the hypotheses of* *Theorem 1.5.* *Let* $0\leq \lambda_{*}<1$ *denote the greatest eigenvalue of* P *other than* *λ* = 1. *Then for any initial state* $G_{0}=\left(V,E_{0}\right)$*,* *we have*

[2] $$\begin{array}{c} \|P^{t}\left(E_{0},\cdot \right){-\;\pi \|}_{\mathrm{TV}}\leq e^{-c} \end{array}$$

*provided* $t\geq \frac{m\log\;2+c}{1-\lambda_{*}}$.

*Theorem* 1.6 is proved in Section 5.

This paper is organized as follows. Section 2 contains notation, basic definitions, and the derivation of the stationary distribution for the evolving graph process using simple edits. In Section 3, we briefly mention results in the theory of left regular bands and establish properties of our graph edit semigroup. In Section 4, we associate the simple edit process to random walks on the chambers of the semigroup, and characterize the eigenvalues of its transition probability matrix. This allows us to give bounds on the mixing time of the process. In Section 5, we consider extensions of this setup to evolving graph processes based on compound edits and investigate two examples of compound edit processes: the Moran forest model and a dynamic random intersection graph model. In Section 6, we provide a formula for the eigenvectors of the transition probability matrix of the simple edit process and a spectral formula for the commute time between subgraphs of the host graph. Section 7 includes some discussion and remarks.

## 2. Basic Properties of the Simple Edit Process

Below we establish some basic properties of the simple edit process introduced in *Definition* 1.1.

Lemma 2.1. *Let* H=(V,E) *be a given host graph, and let* $p={\left(p_{e}\right)}_{e\in E}$ *be given satisfying* $0<p_{e}<1$*.* *Then the simple edit process* ${\left(G_{t}\right)}_{t\geq 0}$ *satisfies the following properties:*

(i) ${\left(G_{t}\right)}_{t\geq 0}$ *is irreducible and aperiodic,*
(ii) ${\left(G_{t}\right)}_{t\geq 0}$ *has a unique stationary distribution given by*
  [3] $$\begin{array}{c} \pi \left(E\right)=\left(\prod_{e\in E}p_{e}\right)\left(\prod_{e\in E\setminus E}\left(1-p_{e}\right)\right), \end{array}$$
(iii) *and* ${\left(G_{t}\right)}_{t\geq 0}$ *is reversible.*

***Proof***: Claim *(i)* follows upon inspection. We establish *(ii)* by direct calculation and show πP=π, where P is the transition probability matrix for ${\left(G_{t}\right)}_{t\geq 0}$ and *π* is as in Eq. **3**. To this end, fix $F\in 2^{E}$, and for any e∈E, let $e^{+}F$ (resp. $e^{-}F$) denote the set obtained by adding (resp. deleting) edge *e* to (resp. from) *F*. We first note that, based upon the definition of ${\left(G_{t}\right)}_{t\geq 0}$, the entry of πP corresponding to *F* can be expressed as follows:

$$\begin{array}{cc} \left(\pi P\right)\left(F\right) & =\sum_{E\in 2^{E}}\pi \left(E\right)P_{\mathrm{EF}}\\ & =\pi \left(F\right)P_{\mathrm{FF}}+\sum_{e\in F}\pi \left(e^{-}F\right)\frac{p_{e}}{m}+\sum_{e\notin F}\pi \left(e^{+}F\right)\frac{1-p_{e}}{m}. \end{array}$$

The transition probability from *F* to itself is given by the sum of the probabilities of adding (resp. removing) each edge which occurs (resp. does not occur) in *F* already, i.e., we have the following formula for the matrix entry $P_{\mathrm{FF}}$:

$$\begin{array}{cc} P_{\mathrm{FF}} & =\sum_{e\in F}\frac{p_{e}}{m}+\sum_{e\notin F}\frac{1-p_{e}}{m}. \end{array}$$

Thus we have

[4] $$\begin{array}{cc} \left(\pi P\right)\left(F\right) & =\sum_{e\in F}\left\{\pi \left(e^{-}F\right)\frac{p_{e}}{m}+\pi \left(F\right)\frac{p_{e}}{m}\right\}\\ & +\sum_{e\notin F}\left\{\pi \left(F\right)\frac{1-p_{e}}{m}+\pi \left(e^{+}F\right)\frac{1-p_{e}}{m}\right\}. \end{array}$$

with the convention that $\sum_{\emptyset }\left(\cdot \right)=0$. Next, we observe from Eq. **3** that it holds $\pi \left(e^{-}F\right)=\frac{1-p_{e}}{p_{e}}\pi \left(F\right)$ whenever *e* ∈ *F* and that $\pi \left(e^{+}F\right)=\frac{p_{e}}{1-p_{e}}\pi \left(F\right)$ if *e* ∉ *F*. Thus, by substitution into Eq. **4**, we have:

$$\begin{array}{cc} \left(\pi P\right)\left(F\right) & =\sum_{e\in F}\frac{\pi \left(F\right)}{m}+\sum_{e\notin F}\frac{\pi \left(F\right)}{m}=\pi \left(F\right). \end{array}$$

Since *(i)* guarantees the uniqueness of any stationary distribution, the claim follows. The proof of *(iii)* follows from a similar calculation.

In the setting where H is the complete graph *K*_*n*_, we have the following examples which show that many edge-independent random graph models can be realized as the stationary distributions of the simple edit process for various choices of ${\left(p_{e}\right)}_{e\in E}$.

Example 2.2 (Erdős-Rényi graphs): Recall that an Erdős-Rényi random graph G(n,p) is constructed by starting with a vertex set V of cardinality *n* and independently adding edge e∈V2 to a graph *G* with probability p∈(0,1), for each such *e*. The stationary distribution of the simple edit process on *K*_*n*_ with edge probabilities $p_{e}=p$ for each e∈V2 is equal to the distribution of G(n,p) on 2V2.

Example 2.3 (Chung-Lu graphs): The Chung-Lu expected degree model, first introduced in ref. 33, is defined as follows. Fix a vertex set V and a sequence of positive real numbers ${\left(k_{v}\right)}_{v\in V}$ with $\max_{v\in V}k_{v}\leq {\left(\sum_{v\in V}k_{v}\right)}^{1/2}$, which serves as the expected degree sequence. Starting with no edges, for each pair e={u,v} with u,v∈V, add the edge *e* to the graph with probability

$$\begin{array}{c} p_{\mathrm{uv}}=\frac{k_{u}k_{v}}{\sum_{w\in V}k_{w}}, \end{array}$$

independently of all other edges. Letting $p={\left(p_{\mathrm{uv}}\right)}_{u,v\in V}$, the stationary distribution of the simple edit process on *K*_*n*_ with edge probabilities p is equal in distribution to the Chung-Lu model.

Example 2.4 (Stochastic Block Model): We consider the setting of *Example* 2.2 in which the probabilities of adding pairs {u,v}∈V2 are inhomogeneous in the following sense. Letting A,B⊆V form a disjoint, nonempty partition of the vertex set V, define

$$\begin{array}{cc} p_{\mathrm{uv}} & =\left\{\begin{array}{cc} p & \text{if}\{u,v\}\subseteq A\;\text{or}\;\{u,v\}\subseteq B\\ q & \text{otherwise,} \end{array}\right. \end{array}$$

for $\left\{u,v\right\}\in \left(\begin{array}{c} V\\ 2 \end{array}\right)$ and constants *p*, *q* satisfying 0<p,q<1. Letting p=(pe)e∈V2, the stationary distribution of the simple edit process on *K*_*n*_ with edge probabilities p is equal in distribution to the two-community stochastic block model.

## 3. Left Regular Bands and the Graph Edit Semigroup

In this section, we state a number of useful facts about the spectral theory for semigroups, and then apply them to the graph edit semigroup. Our notation and terminology on semigroups largely follow ref. 11. The proofs of each of the lemmas appearing in this section are generally straightforward and are omitted.

Recall that a semigroup Σ=(Σ,·) is a set Σ equipped with a binary associative operation · under which it is closed (and which we denote simply by concatenation of elements). We say that Σ is a band if each element is idempotent, i.e., $x^{2}=x$ for each x∈Σ. We say that Σ is a left regular band (LRB) if it is a band and satisfies the memoryless property, i.e., for each x,y∈Σ, xyx=xy holds. These two properties allow one to easily simplify long products of the form $x_{1}x_{2}\cdots x_{n}$ that may arise in Σ by deleting terms in the product which occur more than once on the right. Moreover, it follows that Σ is finite if it is finitely generated.

Definition 3.1 (Graph edit semigroup): Let H=(V,E) be a fixed host graph and let E⊆E be a subset of edges. For each e∈E, let the symbol $e^{+}$ denote the operation of “add edge *e* to *E*” and let $e^{-}$ denote the operation of “remove edge *e* from *E*”. We call these simple edits. We write, for $e_{i}\in E$ and $\sigma_{i}\in \left\{\pm \right\}$,

[5] $$\begin{array}{c} e_{k}^{\sigma_{k}}e_{k-1}^{\sigma_{k-1}}\cdots e_{1}^{\sigma_{1}}E \end{array}$$

to represent the graph obtained by first performing the edit $e_{1}^{\sigma_{1}}$, then so on, until performing the edit $e_{k}^{\sigma_{k}}$. If *x*, *y* are sequences of edits such as those applied to *E* in Eq. **5**, we write *x* = *y* if xE=yE for each E⊆E. The semigroup S=S(H) generated by the set of simple edits is called the graph edit semigroup. By convention, S contains an identity edit denoted $\hat{0}$.

Next we observe that S is a left regular band.

Lemma 3.2. *The graph edit semigroup* S *is a left regular band.*

We note that each x∈S may be written in the form

$$\begin{array}{c} x=e_{k}^{\sigma_{k}}e_{k-1}^{\sigma_{k-1}}\cdots e_{1}^{\sigma_{1}}, \end{array}$$

for some finite collection of simple edits ${\left\{e_{i}^{\sigma_{i}}\right\}}_{i=1}^{k}$, where $e_{i}\in E$ and $\sigma_{i}\in \left\{+,-\right\}$ for each 1≤i≤k. We refer to this as an *edit enumeration of* *x*, and among all such enumerations, those with minimal length as *reduced edit enumerations ofx*. If *e* ≠ *f* in E and σ,τ∈{±}, then naturally $e^{\sigma }f^{\tau }=f^{\tau }e^{\sigma }$ so that distinct edges may appear anywhere in the sequence. Consequently, we observe that any such reduced edit enumeration must contain at most one element of the form $e^{\left(\cdot \right)}$ for any particular e∈E, since $e^{+}e^{-}=e^{+}$ and $e^{-}e^{+}=e^{-}$ for any e∈E. Therefore, each reduced edit enumeration of a given element x∈S contains the same set of simple edits, possibly appearing in a different order. Using these facts it is straightforward to show that the cardinality of S is $3^{m}$. For x∈S, we write simple(x) for the set of simple edits obtained from any reduced edit enumeration of *x*.

For a generic LRB Σ, we can equip Σ with the relation ⩽ defined by *x* ⩽ *y* if xy=y. It is relatively straightforward to show that ⩽ is transitive (for any semigroup) and reflexive (since Σ is a band); moreover, ⩽ is antisymmetric and thus defines a partial order on Σ owing to the memorylessness property (see ref. 11, proposition 7). In the case of the graph edit semigroup S, this partial order can be nicely understood in terms of the edges affected by particular edits.

Lemma 3.3. *Let* *x* *and* *y* *be two fixed elements of the graph edit semigroup* S*.* *Then* *x* *⩽* *y* *if and only if* simple(x)⊆simple(y)*.*

We can equip a generic LRB Σ with the further relation ≺ by writing *x* ≺ *y* if yx=y. It is straightforward to show that ≺ is transitive and reflexive (in the latter case, since Σ is a band), but in general not antisymmetric and therefore not a partial order. Define *x* ≃ *y* whenever *x* ≺ *y* and *y* ≺ *x* and set L=Σ/≃. Then *L* is a partially ordered set under ≺ and if we denote by supp:Σ→L the quotient map, we have that *x* ≺ *y* holds if and only if supp x≺supp y.

Since xy≻x and xy≻y for each x,y∈Σ (because of the memoryless and idempotence properties, respectively), we have that *L* is a join semilattice, and moreover, it can be shown that for each x,y∈Σ, we have

[6] $$\begin{array}{c} \mathrm{supp}\;\mathrm{xy}=\mathrm{supp}\;x\cup \mathrm{supp}\;y. \end{array}$$

Elements *X* ∈ *L* are called *flats* in the hyperplane arrangement literature (see, e.g., ref. 10). If Σ has an identity element (and therefore *L* has a minimal element) and if *L* is finite, then *L* will be a true lattice (see, e.g., ref. 32, section 3.3). The following lemma completely characterizes this lattice in the case of the graph edit semigroup S.

Lemma 3.4. *Let* *x* and *y* *be two fixed elements of the graph edit semigroup* S*.* *Then* *x* *≺* *y* *if and only if* supp x⊆supp y*.* *The corresponding lattice* L=S/≃ *can be identified as the Boolean algebra of subsets of edges* E*.*

Remark 3.5: It follows from the proof of *Lemma* 3.4 that the quotient map supp:S→L can be realized as the map which sends each edit to the subset of E consisting of edges on which the edit acts in a nontrivial manner.

We say that an element c∈Σ is a *chamber* provided that cx=c for each x∈Σ, or equivalently, *c* is maximal in the partially ordered set (Σ,⩽). The set of chambers in an LRB Σ forms a two-sided ideal (see, e.g., ref. 11, Proposition 9). We denote by C the ideal of chambers in the graph edit semigroup S. The following lemma characterizes chambers in this setting.

Lemma 3.6. *An element* *c* *in the graph edit semigroup* S *is a chamber if and only if* supp c=E*.* *In turn, by identifying* *c* *with the set of edges* e∈E *such that* $e^{+}\in simple\left(c\right)$*,* *each chamber may be identified as a subset* $E\in 2^{E}$ *in a one-to-one and onto fashion.*

We remark that, in light of *Lemma* 3.6, a random walk on the chambers of S may be identified as a random walk on labeled subgraphs of H. Moreover, *Lemma* 3.6 leads to a bijection between the chambers of S and the associated lattice L via the maps described in *Lemmas* 3.4 and 3.6. In Section 5, we consider the case of semigroups generated by compound edits and random walks on their chambers.

## 4. Eigenvalues and Mixing Times of the Simple Edit Process

For a LRB *S* and a probability measure $w\in R^{S}$, we define the semigroup random walk according to the transition probability matrix *P*_*S*_ given by

[7] $$\begin{array}{cc} P_{S}\left(x,y\right) & =\sum_{\begin{array}{c} z\in S\\ zx=y \end{array}}w_{z},\;x,y\in S \end{array}$$

Notice that if *C* is the set of chambers in *S*, then for each *c* ∈ *C*, $P_{S}\left(c,x\right)>0$ only when there exists *z* ∈ *S* such that $w_{z}>0$ and zc=x. But *C* is a left ideal so zc∈C and thus *x* ∈ *C* as well. In other words, if and when the random walk reaches the ideal of chambers, it will remain in the ideal for all time. Therefore, rather than studying a Markov chain on the state space *S* we instead use as a state space the chambers *C*. We note that the transition probability matrix *P*_*C*_ associated to the semigroup random walk on *C* is the square submatrix of *P*_*S*_ indexed by the elements of *C*:

[8] $$\begin{array}{c} P_{C}={\left(P_{S}\left(x,y\right)\right)}_{x,y\in C}. \end{array}$$

Our main result in this section characterizes the eigenvalues of the transition probability matrix P corresponding to the simple edit process introduced in *Definition* 1.1. We note that Theorem 1.2 follows from the following.

Theorem 4.1. *Let* S=S(H) *denote the graph edit semigroup corresponding to the host graph* H *as in* *Definition 3.1.* *Let* ${\left(w_{x}\right)}_{x\in S}$ *be a probability distribution on* S*,* *and let* $P_{C}$ *be the Markov transition probability matrix for the random walk on the chambers* C *of the graph edit semigroup* S *(as in Eq.* ***8****).* *Then* $P_{C}$ *is diagonalizable and has an eigenvalue with multiplicity one for each subset* T⊆E *given by*

[9] $$\begin{array}{c} \lambda_{T}=\sum_{\begin{array}{c} y\in S\\ \mathrm{supp}\;y\subseteq T \end{array}}w_{y}. \end{array}$$

*In the special case where* ${\left(w_{x}\right)}_{x\in S}$ *is nonzero exactly on the set of simple edits with edit probabilities determined by* $p={\left(p_{e}\right)}_{e\in E}$ *such that* $0<p_{e}<1$ *and which satisfies*

$$\begin{array}{c} w_{e^{+}}=\frac{p_{e}}{m},\;w_{e^{-}}=\frac{1-p_{e}}{m}, \end{array}$$

*then the random walk on* C *has the same transition probability matrix as the simple edit process with edge probabilities* p*.* *Moreover, its eigenvalues are indexed by subsets* T⊆E *and for each such subset we have*

[10] $$\begin{array}{c} \lambda_{T}=\frac{\left|T\right|}{m}, \end{array}$$

*independent of* ${\left(p_{e}\right)}_{e\in E}$*.*

Remark 4.2: We remark that the eigenvalue *λ*_*T*_ in *Theorem* 4.1 that is *indexed by* T⊆E occurs with multiplicity $m_{T}=1$, but distinct *T* may give rise to the same value λ∈R. In the case of the simple edit process, each eigenvalue of the form $\frac{k}{m}$ for some 0≤k≤m occurs with multiplicity mk.

***Proof of Theorem 4.1:*** The main tool that we use here is (11, theorem 1), from which it follows that the eigenvalues are as in Eq. **9**, each with multiplicity *m*_*X*_ satisfying

$$\begin{array}{c} \sum_{\begin{array}{c} Y\in L\\ Y≻X \end{array}}m_{Y}=c_{X}, \end{array}$$

where $c_{\mathrm{supp}\;x}=\left|\left\{y\in C:y⩾x\right\}\right|$. Alternatively,

$$\begin{array}{c} m_{X}=\sum_{\begin{array}{c} Y\in L\\ Y\geq X \end{array}}\mu \left(X,Y\right)c_{Y}, \end{array}$$

where *μ* is the Möbius function of *L* (see ref. 32, section 3.7). We claim that the multiplicity of each eigenvalue *λ*_*T*_ is equal to one. To this end, start by assuming that |T|=m, i.e., T=E. Recall that the set of chambers C of S can be identified as consisting of edits whose reduced edit enumerations contain simple edits whose edges exhaust E. Thus we have that by *Lemma* 3.3 and (11, theorem 1), letting x∈S be any element with supp x=E (i.e., x∈C),

$$\begin{array}{cc} c_{E=\mathrm{supp}\;x} & =|\left\{y\in C:simple(y)⊇simple(x)\right\}|=1. \end{array}$$

since the only other chamber whose reduced edit enumeration subsumes that of a chamber *x* is *x* itself. Therefore, $m_{E}$ satisfies

$$\begin{array}{c} 1=\sum_{\begin{array}{c} T^{\prime }\in L\\ T^{\prime }⊇E \end{array}}m_{T^{\prime }}=m_{E}. \end{array}$$

Next, assume for some 1≤k≤m that $m_{Q}=1$ holds for each Q⊆E of size k≤Q≤m and let T⊆E be any fixed subset such that |T|=k−1. Then we have for any x∈S such that supp x=T,

$$\begin{array}{cc} c_{T} & =|\left\{y\in C:simple(y)⊇simple(x)\right\}|=2^{m-(k-1)} \end{array}$$

since there are m−(k−1) edges not belonging to supp x, and the chambers in question may be enumerated by concatenating to a reduced edit enumeration of *x* products of simple edits for each of the remaining edges with any choice of sign. Therefore, *m*_*T*_ satisfies

$$\begin{array}{c} 2^{m-(k-1)}=\sum_{\begin{array}{c} T^{\prime }\in L\\ T^{\prime }⊇T \end{array}}m_{T^{\prime }}=m_{T}+\sum_{\begin{array}{c} T^{\prime }\in L\\ T^{\prime }⊋T \end{array}}m_{T^{\prime }}. \end{array}$$

By the induction assumption, we have that $m_{T^{\prime }}=1$ for each $T^{\prime }⊇T$, and therefore it follows that

$$\begin{array}{c} m_{T}+\sum_{\begin{array}{c} T^{\prime }\in L\\ T^{\prime }⊋T \end{array}}m_{T^{\prime }}=m_{T}+2^{m-(k-1)}-1, \end{array}$$

leaving $m_{T}=1$ as a result. Our claim that $m_{T}=1$ for each *T* follows. For the second half of the claim, we can recognize the semigroup random walk on the chambers of S as the simple edit process introduced in Section 2 by identifying a chamber c∈C with

$$\begin{array}{c} E_{c}=\left\{e\in E:e^{+}\in simple\left(c\right)\right\}\subseteq E. \end{array}$$

Finally, in this case, if T⊆E, we have by ref. 11, theorem 1 that

$$\begin{array}{cc} \lambda_{T} & =\sum_{\begin{array}{c} y\in S\\ \mathrm{supp}\;y\subseteq T \end{array}}w_{y}=\sum_{e\in T}\left(w_{e^{+}}+w_{e^{-}}\right)\\ & =\sum_{e\in T}\left\{\frac{p_{e}}{m}+\frac{1-p_{e}}{m}\right\}=\frac{\left|T\right|}{m}. \end{array}$$

The second claim follows.

Next, we use the eigenvalues of the simple edit process to deduce bounds on its mixing time. We begin by recalling that if $\mu ,\nu \in R^{X}$ are probability distributions on a state space X, their total variation distance is given by

[11] $$\begin{array}{cc} {\left\|\mu -\nu \right\|}_{\text{TV}} & :=\;\max_{A\subseteq X}\left|\sum_{x\in A}\mu \left(x\right)-\nu \left(x\right)\right|=\frac{1}{2}\sum_{x\in X}\left|\mu \left(x\right)-\nu \left(x\right)\right|. \end{array}$$

If ${\left(X_{t}\right)}_{t\geq 0}$ is a Markov chain on a finite state space X with stationary distribution *π*, its mixing time is the least time step *t* after which $\|P^{t}{\left(y,\cdot \right)-\pi \|}_{\text{TV}}$ is below some fixed cutoff for each y∈X. In the case of a random walk on the chambers of a left regular band, we recall the following specialized result on the rate of convergence to stationarity.

Theorem 4.3 [Theorem 0, (11)]. *Let* *S* *be a finite LRB with identity and associated lattice* *L*. *Let* ${\left\{w_{x}\right\}}_{x\in S}$ *be a probability distribution on *S* such that *S* is generated by* $\left\{x\in S:w_{x}>0\right\}$*. Then the random walk on the ideal* *C* *of chambers of* *S* *(defined by the transition probabilities in* *Eq.* **8***) has a unique stationary distribution* *π,* *and the total variation distance of the walk from stationarity after starting at an initial chamber* $x_{0}\in C$ *satisfies, for each* *t* *>* *0*,

$$\begin{array}{c} \|P_{C}^{t}\left(x_{0},\cdot \right){-\;\pi \|}_{\mathrm{TV}}\leq \sum_{X\in L^{*}}m_{X}\lambda_{X}^{t}, \end{array}$$

*where* $L^{*}$ *consists of each except the maximal element of* *L* *and* *λ*_*X*_ *(resp.* *m*_*X*_*) denotes the eigenvalue (resp. the multiplicity) of the element* $X\in L^{*}$*,* *as in (*11*, theorem 1).*

We note that a more general version of this theorem appears in ref. 9, and the result itself appears first in the hyperplane walk literature (10). We can apply this directly to the case of the simple edit process, as follows, to establish that the graph edit process mixes in time near-linear in *m*. We note that *Theorem* 1.3 follows from the result below.

Theorem 4.4 *Let* H=(V,E) *be a fixed host graph and let* $p={\left(p_{e}\right)}_{e\in E}$ *be fixed with* $0<p_{e}<1$ *for each* e∈E*.* *Let* ${\left(G_{t}\right)}_{t\geq 0}$ *be obtained from the simple edit process with edge probabilities* p*. Let* P *denote the corresponding transition probability matrix and let* *π* *denote its stationary distribution. Then, for each* t≥2mlogm *and initial state* $G_{0}=\left(V,E_{0}\right)$*,* *we have*

[12] $$\begin{array}{c} \|P^{t}\left(E_{0},\cdot \right){-\;\pi \|}_{\mathrm{TV}}\leq 2m{\left(1-\frac{1}{m}\right)}^{t}. \end{array}$$

*In particular, if* *c* *>* *0 is given, we have*

$$\begin{array}{c} \|P^{t}\left(E_{0},\cdot \right){-\;\pi \|}_{\mathrm{TV}}\leq e^{-c} \end{array}$$

*provided* t≥m(c+2logm).

***Proof:*** By using *Theorems* 4.1 and 4.3, we have

‖Pt(E0,·)−π‖TV≤∑X∈L∗mXλXt≤∑k=1m−1kmtmk

Let ak=kmtmk, then we have for *k* ≥ 1,

$$\begin{array}{cc} \frac{a_{k}}{a_{k+1}} & =\frac{k^{t}}{{\left(k+1\right)}^{t}}\frac{\left(k+1\right)}{m-k}\leq m{\left(1-\frac{1}{m}\right)}^{t}\leq me^{-t/m} \end{array}$$

Thus if t≥2mlogm, then $me^{-t/m}\leq \frac{1}{m}\leq 1/2$. Therefore, we have that

$$\begin{array}{c} a_{k}\leq {\left(\frac{1}{2}\right)}^{m-1-k}a_{m-1}, \end{array}$$

so that in turn

∑k=1m−1kmtmk≤m1−1mt∑k=1m−112m−1−k≤2m1−1mt

and Eq. **12** is proved. Note that

$$\begin{array}{c} 2m{\left(1-\frac{1}{m}\right)}^{t}\leq e^{-c} \end{array}$$

holds provided

$$\begin{array}{cc} t & \geq -\frac{c+\;\log\;2m}{\log\;1\;-\;1/m}. \end{array}$$

Since log(1+x)≤x for x>−1, we have

$$\begin{array}{cc} -\frac{c\;+\;\log\;2m}{\log\;1\;-\;1/m} & \leq m(c+\log2m)\leq m(c+2\log m), \end{array}$$

where the last estimate shows t≥2mlogm implies the bound Eq. **12**. The theorem is proved.

## 5. Compound Edit Processes and the Moran Forest

In this section, we consider the setting of compound edit processes, which were introduced in *Definition* 1.4. In this setting, the resulting evolving processes are considerably more complex when compared to the simple edit process considered in earlier sections. Nevertheless, our methods can be used to obtain closed-form formulas for the eigenvalues of the transition probability matrices as well as bounds for the corresponding Markov chain mixing times. We consider two examples of compound edit processes which are motivated by previous work in population biology modeling and random graphs, respectively, and compute their eigenvalues. We remark that our methods can be used to analyze further processes such as population dynamic models, birth–death processes, or contact-diffusion models.

Elements of the graph edit semigroup S are called compound edits, which are products of simple edits. Thus compound edit processes can be viewed as random walks on the chambers of subsemigroups of S which are generated by specified families of compound edits and we may thereby apply much of the setup from Section 3. We note that the chambers of semigroups generated by compound edits will form a subset of the chambers of the larger graph edit semigroup. The following lemma characterizes the join semilattice associated to such subsemigroups.

Lemma 5.1. *Let* A⊆S *be a given set of compound edits belonging to the graph edit semigroup* S *and let* T=⟨A⟩ *be the subsemigroup of* S *generated by the edits* A*. Then the join semilattice* L(T) *associated to* T *(as in* *Lemma* *3.4**)* *is generated by the sets* ${\left\{\mathrm{supp}\;\left(x\right)\right\}}_{x\in A}$ *and the set union operation.*

Equivalently, L(T) may be described as the smallest subset of $2^{E}$ containing the sets $\emptyset ,{\left\{\mathrm{supp}\;\left(x\right)\right\}}_{x\in A}$ and which is closed under the map X,Y↦X∪Y. The proof of this result is essentially identical to the proof of *Lemma* 3.4 and is omitted. Next we characterize the eigenvalues of the random walk on the chambers of a subsemigroup T as in *Lemma* 5.1.

Theorem 5.2. *Let* H *denote a fixed host graph and let* A⊆S *be a given set of edits, let* $w\in R^{A}$ *be a given probability distribution on* A*, and let* T=⟨A⟩ *be the subsemigroup of* S *generated by the edits* A*, and which by convention contains the identity edit* $\hat{0}$*. Consider the random walk on the chambers* C *of* T *with transition probability matrix* $P_{C}$ *(as in Eq.* ***8****). Then* $P_{C}$ *coincides with the transition probability matrix of the compound edit process obtained from* A *with distribution* *w.* *Moreover,* $P_{C}$ *is diagonalizable and has eigenvalues indexed by elements* X∈L(T)*, each of which is identified as a subset of* E*, with corresponding eigenvalue*

$$\begin{array}{c} \lambda_{X}=\sum_{\begin{array}{c} x\in A\\ \mathrm{supp}\;(x)\subseteq X \end{array}}w_{x}. \end{array}$$

*The multiplicity of* *λ*_*X*_ *depends on the choice of* A *and can be computed from the Möbius function of the join semilattice generated by* ${\left\{\mathrm{supp}\;\left(x\right)\right\}}_{x\in A}$ *and set union (see refs.* *11**, theorem 1 and* *32**, section 3.7).*

We note that the number of eigenvalues of $P_{C}$ and their multiplicities depend significantly on the choice of generating family A and thus do not admit a general closed-form enumeration. The proof is essentially the same as the first half of that of *Theorem* 4.1 and is omitted.

By *Theorem* 4.3, we know that the random walk on the chambers of T will be ergodic and its mixing time can be bounded by the eigenvalues *λ*_*X*_ of $P_{C}$. Without the multiplicities of the eigenvalues, our mixing time bounds (cf. *Theorem* 4.4) are different, but we can still guarantee a mixing time which is polynomial in *m* provided each of the weights *w*_*x*_ in *Theorem* 5.2 are bounded from below by the reciprocal of a polynomial in *m*. We make this precise below.

Theorem 5.3. *Let* A⊆S *be a given family of edits, let* T=⟨A⟩ *be the subsemigroup of* S *generated by* A*,* *let* $w\in R^{A}$ *be a given probability distribution on* A*,* *and let*

$$\begin{array}{c} \lambda_{*}=\underset{\begin{array}{c} X\in L(T)\\ X\neq E \end{array}}{\sup}\lambda_{X} \end{array}$$

*as in* *Theorem* *5.2.* *Consider the compound edit process* ${\left(G_{t}\right)}_{t\geq 0}$ *obtained from* A *with distribution* *w,* *and let* P *denote its transition probability matrix and let *π* denote its stationary distribution. Then for any initial state* $G_{0}=\left(V,E_{0}\right)$ *belonging to the chambers of* T *and* *c* *> 0, we have*

[13] $$\begin{array}{c} \|P^{t}\left(E_{0},\cdot \right){-\;\pi \|}_{\mathrm{TV}}\leq e^{-c} \end{array}$$

*provided* $t\geq \frac{m\;\log\;2+c}{1-\lambda_{*}}$.

***Proof:*** By *Theorem* 4.3, we have that

$$\begin{array}{c} \|P^{t}\left(E_{0},\cdot \right){-\pi \|}_{\mathrm{TV}}\leq \sum_{X\in L^{*}}m_{X}\lambda_{X}^{t}. \end{array}$$

We bound the right-hand side as follows. First, the largest eigenvalue *λ*_*X*_ must be at most $\lambda_{*}$; and second, the number of chambers of T is at most the number of chambers of the entire graph edit semigroup S, which is $2^{m}$. Thus

$$\begin{array}{cc} \sum_{X\in L^{*}}m_{X}\lambda_{X}^{t} & \leq 2^{m}\;\lambda_{*}^{t}. \end{array}$$

Therefore, by straightforward manipulation, we have that $2^{m}\;\lambda_{*}^{t}\leq e^{-c}$ if and only if

$$\begin{array}{c} t\geq -\frac{m\;\log\;2\;+\;c}{\log(\lambda_{*})}, \end{array}$$

and thus by using the bound log1−x≤−x for x>−1, we have that

$$\begin{array}{cc} -\frac{m\;\log\;2\;+\;c}{\log(\lambda_{*})} & \leq \frac{m\;\log\;2\;+\;c}{1-\lambda_{*}}, \end{array}$$

from which the claim follows.

Remark 5.4: If the cardinality of the set of chambers of the semigroup T in *Theorem* 5.3 can be bounded from above by *M* ≥ 0, Eq. **2**,**13** can be sharpened to $t\geq \frac{\log\;M+c}{1-\lambda_{*}}$.

To conclude this section, we consider two examples of compound edit processes, the Moran forest model and a dynamic random intersection graph model.

**Fig. 4.**
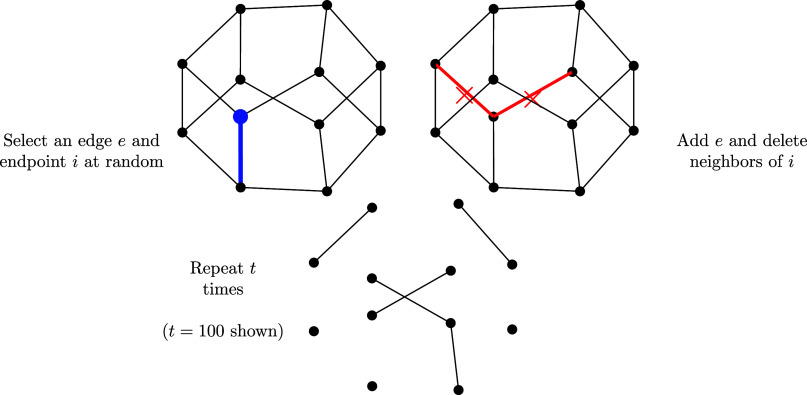
An illustration of the Moran edit process with host graph H on twelve vertices.

Example 5.5 (The Moran forest model): Let H=(V,E) be a given host graph and set $G_{0}=H$. For each *t* ≥ 0, sample an edge e={u,v}∈E uniformly at random and then an endpoint x∈{u,v} uniformly at random. Let $G_{t+1}=\left(V,E_{t+1}\right)$ be obtained by deleting all edges from *E*_*t*_ which are incident to the node *x* and adding the edge *e* to *E*_*t*_. If ${\left(G_{t}\right)}_{t\geq 0}$ is a sequence of random graphs obtained from this model, we say ${\left(G_{t}\right)}_{t\geq 0}$ is obtained from the Moran edit process. The stationary distribution *π* obtained from $H=K_{n}$ is known as the Moran forest model (7). We illustrate an example of this Markov chain in Fig. 4.

It can be shown without any semigroup theory that this process is ergodic and thus has a unique stationary distribution (see, e.g., ref. 7); it has been used as a model for evolutionary processes and in particular can be used to model the family structure of a random population obtained from the Moran model (see ref. 34). The state graph associated to this walk in the case where $H=K_{4}$ is shown in Fig. 2.

We can recast this process as a particular instance of an edit semigroup random walk as follows. We begin by letting $E^{\prime }=\left\{\left(u,v\right),\left(v,u\right):\left\{u,v\right\}\in E\right\}$ denote the oriented edges of the host graph H. For each $\left(u,v\right)\in E^{\prime }$, write

$$\begin{array}{c} y_{\left(u,v\right)}={\left\{u,v\right\}}^{+}\left(\prod_{\begin{array}{c} e\in E\\ u\in e \end{array}}e^{-}\right), \end{array}$$

where we use the notation ${\left\{u,v\right\}}^{+}$ to refer to the edit which adds the edge {u,v} to a given subgraph. We define

$$\begin{array}{c} B=\{y_{\left(u,v\right)}:\left(u,v\right)\in E^{\prime }\}, \end{array}$$

and denote by M=⟨B⟩ the semigroup which is generated by B. In the setup of *Theorem* 5.2, by defining for each v∈V the set N(v):={e∈E:v∈e}, it follows by *Lemma* 5.1 that the join semilattice L(M) is the smallest subset of $2^{E}$ containing the neighborhoods ${\left\{N\left(v\right)\right\}}_{v\in V}$ and *∅* and which is closed under set unions. Moreover, by *Theorem* 5.2, it follows that the eigenvalues *λ*_*X*_ of the transition probability matrix P of the Moran edit process are indexed by subsets X∈L(M) and are given by

$$\begin{array}{c} \lambda_{X}=\sum_{\begin{array}{c} v\in V\\ N(v)\subseteq X \end{array}}\frac{d_{v}}{2m}, \end{array}$$

where *d*_*v*_ denotes the degree of vertex v∈V in the host graph. It follows that the second largest eigenvalue of P is at most $1-\frac{\delta (H)}{m}$, where δ(H) is the minimum vertex degree of H. By *Theorem* 5.3,

$$\begin{array}{c} \|P^{t}\left(G_{0}=H,\cdot \right){-\;\pi \|}_{\mathrm{TV}}\leq e^{-c} \end{array}$$

holds provided

[14] $$\begin{array}{c} t\geq \frac{m^{2}\;\log\left(2\right)+cm}{\delta (H)}. \end{array}$$

In the case where $H=K_{n}$ for some *n* ≥ 2, by *Remark* 5.4, Eq. **14** can be sharpened using the bound $F_{n}\leq {\left(n-1\right)}^{n}\leq e^{n\;\log\;n}$, where *F*_*n*_ is the number of spanning forests of *K*_*n*_ (see ref. 35, theorem 1.4), so that it suffices to pick

[15] $$\begin{array}{c} t\geq \frac{n^{2}\;\log\;n\;+\;cn}{2}. \end{array}$$

Example 5.6 (Dynamic random intersection graph): Consider a set of *n* ≥ 1 ground symbols Ω={1,2,⋯,n}. An intersection graph has vertex set *V* which is a family of subsets of Ω and edges {u,v} whenever u∩v≠∅. An intersection graph is also understood via its bipartite incidence graph, which consists of vertex set Ω∪V with edges {v,B} for v∈Ω and *B* ∈ *V* present whenever *v* ∈ *B*.

Various examples of random intersection graphs have appeared in the literature dating back to the 1996 thesis of Singer (36). Here, we consider a dynamic random intersection graph model [not to be confused with (37)] with stationary distribution equal to the random intersection graph model of Godehardt and Jaworski (8), as follows.

Let $\Omega^{\prime }=\left\{1,2,\cdots ,N\right\}$ for *N* ≥ 1 be fixed. Let the host graph H be the complete bipartite graph on $\Omega \cup \Omega^{\prime }$. For v∈Ω and $A\subseteq \Omega^{\prime }$ fixed, define the compound graph edit

$$\begin{array}{cc} y_{v,A} & =\prod_{\begin{array}{c} u\in \Omega^{\prime }\\ u\in A \end{array}}{\left\{u,v\right\}}^{+}\prod_{\begin{array}{c} u\in \Omega^{\prime }\\ u\notin A \end{array}}{\left\{u,v\right\}}^{-} \end{array}$$

That is, $y_{v,A}$ is the edit which assigns the neighborhood of *v* to consist exactly of the elements of *A*. Note that the support of $y_{v,A}$ consists of the *N* edges in H with endpoint *v*. We consider the set of compound edits $B={\left\{y_{v,A}\right\}}_{v\in \Omega ,A\subseteq \Omega^{\prime }}$.

Next, we fix a probability distribution $\mu \in R^{N+1}$ on the set {0,1,⋯,N} which are understood as the possible cardinalities of neighborhoods of vertices in Ω. Define a probability distribution *w* on B as follows:

$$\begin{array}{cc} w(y_{v,A}) & =\frac{1}{n}\frac{\mu (|A|)}{\left(\begin{array}{c} N\\ |A| \end{array}\right)},\;v\in \Omega ,\;A\subseteq \Omega^{\prime }. \end{array}$$

That is, $w_{v,A}$ may be understood as the measure of a uniformly selected vertex *v* and a subset *A* sampled first by its cardinality according to *μ* and then uniformly among all subsets of $\Omega^{\prime }$ with corresponding cardinality. The compound edit process on the host graph H with edits B and distribution *w* can be thought of as iteratively selecting a vertex v∈Ω at random and then resetting its neighbors in $\Omega^{\prime }$ according to the update rule given by *μ*. It is straightforward to verify that its stationary distribution agrees with the random intersection graph model defined by n,N,μ (see ref. 8).

Assuming *μ* has full support, the set of chambers of the associated subsemigroup I=⟨B⟩ is the same as the entire edit semigroup and has cardinality $2^{\mathrm{nN}}$. Moreover, the eigenvalues of this walk are indexed by elements of the join semilattice generated by the edge neighborhoods of each vertex v∈Ω. Since these sets are in fact disjoint, for each subset B⊆Ω, there is an eigenvalue

$$\begin{array}{c} \lambda_{B}=\sum_{v\in B}\sum_{\begin{array}{c} y\in B\\ \mathrm{supp}\;(y)=N(v) \end{array}}w\left(y\right)=\frac{\left|B\right|}{n}, \end{array}$$

so that the spectral gap is 1/n independent of the choice of *μ*. By *Theorem* 5.3 and *Remark* 5.4, letting P (resp. *π*) denote the transition probability matrix (resp. stationary distribution) for the compound edit process, for any given initial state $G_{0}=\left(\Omega \cup \Omega^{\prime },E_{0}\right)$,

$$\begin{array}{c} \|P^{t}\left(E_{0},\cdot \right){-\;\pi \|}_{\mathrm{TV}}\leq e^{-c} \end{array}$$

holds provided $t\geq Nn^{2}\log2+cn$.

## 6. Eigenvectors of the Graph Edit Markov Chain and Commute Times

In this section, we obtain closed-form expressions for each of the eigenvectors of the transition probability matrix P of the simple edit process. The main tool utilized in discovering the eigenvectors comes from the recursive procedure introduced by Saliola in the paper (12); we mention this approach in order to provide context to the reader, although the argument presented here is self-contained.

Theorem 6.1. *Let* H=(V,E) *be a given host graph and let* P *denote the transition probability matrix of the simple edit process with edge probabilities* $p={\left(p_{e}\right)}_{e\in E}$*.* *Letting* T⊆E *be any subset of edges, define the row vector* $\phi_{T}\in R^{2^{E}}$ *componentwise via the formula*

[16] $$\begin{array}{cc} \phi_{T}\left(E\right) & ={\left(-1\right)}^{\left|E\setminus (E\cup T)\right|}\prod_{e\in T\cap E}p_{e}\prod_{e\in T\setminus E}\left(1-p_{e}\right),\;E\subseteq E. \end{array}$$

Then *ϕ*_*T*_ satisfies $\phi_{T}P=\frac{\left|T\right|}{m}\phi_{T}$.

***Proof:*** We can prove this using a downward induction on the size of the subset *T*. For the base case |T|=|E| we know that the claim is true since $\phi_{E}=\pi$ as in *Lemma* 2.1. Assume the claim is true for each such *T* with |T|≥k, where 1≤k≤|E|, and fix a subset T⊆E with |T|=k−1. We write $T=\{t_{1},\cdots ,t_{k-1}\}$ for $t_{i}\in E$ and put

$$\begin{array}{c} T^{*}=\left\{t_{1},\cdots ,t_{k-1},t_{*}\right\}, \end{array}$$

where $t_{*}\in E$ is any element which does not originally appear in *T*. Thus $T=T^{*}\setminus \left\{t_{*}\right\}$ and for each E⊆E it holds

[17] $$\begin{array}{cc} \left(\phi_{T^{*}}P\right)\left(E\right) & =\frac{|T^{*}|}{m}\phi_{T^{*}}\left(E\right). \end{array}$$

Moreover, we have upon inspection that

[18] $$\begin{array}{cc} \phi_{T}\left(E\right) & =\left\{\begin{array}{cc} \frac{1}{p_{t_{*}}}\phi_{T^{*}}\left(E\right) & \text{if}\;t_{*}\in E\\ -\frac{1}{1-p_{t_{*}}}\phi_{T^{*}}\left(E\right) & \text{if}\;t_{*}\notin E \end{array}\right.. \end{array}$$

Now fix any E⊆E and assume $t_{*}\in E$. We observe that

[19] $$\begin{array}{cc} \phi_{T^{*}}\left(t_{*}^{-}E\right) & =\frac{1-p_{t_{*}}}{p_{t_{*}}}\phi_{T^{*}}\left(E\right). \end{array}$$

We can proceed with the calculation of $\phi_{T}P$ (using an argument along the lines of that in the proof of *Lemma* 2.1) as follows:

$$\begin{array}{cc} \left(\phi_{T}P\right)\left(E\right) & =\sum_{e\in E}\left\{\phi_{T}\left(e^{-}E\right)\frac{p_{e}}{m}+\phi_{T}\left(E\right)\frac{p_{e}}{m}\right\}\\ & +\sum_{e\notin E}\left\{\phi_{T}\left(e^{+}E\right)\frac{1-p_{e}}{m}+\phi_{T}\left(E\right)\frac{1-p_{e}}{m}\right\}\\ & =\frac{1}{p_{t_{*}}}[\sum_{e\in E}\left\{\phi_{T^{*}}\left(e^{-}E\right)\frac{p_{e}}{m}+\phi_{T^{*}}\left(E\right)\frac{p_{e}}{m}\right\}\\ & +\sum_{e\notin E}\left\{\phi_{T^{*}}\left(e^{+}E\right)\frac{1-p_{e}}{m}+\phi_{T^{*}}\left(E\right)\frac{1-p_{e}}{m}\right\}]\\ & +\phi_{T}\left(t_{*}^{-}E\right)\frac{p_{t_{*}}}{m}-\frac{\phi_{T^{*}}\left(t_{*}^{-}E\right)}{p_{t_{*}}}\frac{p_{t_{*}}}{m}\\ & =\frac{|T^{*}|}{mp_{t_{*}}}\phi_{T^{*}}\left(E\right)+\phi_{T}\left(t_{*}^{-}E\right)\frac{p_{t_{*}}}{m}-\frac{\phi_{T^{*}}\left(t_{*}^{-}E\right)}{p_{t_{*}}}\frac{p_{t_{*}}}{m} \end{array}$$

using Eqs. **17** and **18**. Using Eq. **18** again and Eq. **19**, it follows that

$$\begin{array}{cc} & \frac{|T^{*}|}{mp_{t_{*}}}\phi_{T^{*}}\left(E\right)+\phi_{T}\left(t_{*}^{-}E\right)\frac{p_{t_{*}}}{m}-\frac{\phi_{T^{*}}\left(t_{*}^{-}E\right)}{p_{t_{*}}}\frac{p_{t_{*}}}{m}\\ & =\frac{|T^{*}|}{mp_{t_{*}}}\phi_{T^{*}}\left(E\right)-\frac{\phi_{T^{*}}\left(E\right)}{1-p_{t_{*}}}\frac{1-p_{t_{*}}}{p_{t_{*}}}\frac{p_{t_{*}}}{m}\\ & -\frac{\phi_{T^{*}}\left(E\right)}{p_{t_{*}}}\frac{p_{t_{*}}}{m}\frac{1-p_{t_{*}}}{p_{t_{*}}}\\ & =\frac{\phi_{T^{*}}\left(E\right)}{p_{t_{*}}}\left\{\frac{|T^{*}|-p_{t_{*}}-1+p_{t_{*}}}{m}\right\}\\ & =\phi_{T}\left(E\right)\frac{|T^{*}|-1}{m}=\phi_{T}\left(E\right)\frac{\left|T\right|}{m} \end{array}$$

as claimed. The case $t_{*}\notin E$ follows similarly, and thus by induction, the claim follows.

The next result confirms that the family *ϕ*_*T*_ exhibited in *Theorem* 6.1, when suitably normalized, forms an orthonormal family in $R^{2^{E}}$.

Theorem 6.2. *Let* H=(V,E) *be a given host graph and let* P *(resp. *π*)* *denote the transition probability matrix (resp. stationary distribution) of the simple edit process with edge probabilities* $p={\left(p_{e}\right)}_{e\in E}$*.* *For each* T⊆E*,* *let* *ϕ*_*T*_ *denote the corresponding eigenvector of* P *as in Eq.* ***16****.* *Let the row vector* $\psi_{T}\in R^{2^{E}}$ *be given by the formula*

$$\begin{array}{cc} \psi_{T}\left(E\right) & =\phi_{T}\left(E\right)\frac{\prod_{e\notin T}\sqrt{p_{e}\left(1-p_{e}\right)}}{\sqrt{\pi (E)}},\;E\subseteq E. \end{array}$$

*Then* ${\left\{\psi_{T}\right\}}_{T\subseteq E}$ *form an orthonormal system of (left) eigenvectors for the symmetric matrix* $Q=\Pi^{1/2}P\Pi^{-1/2}$*,* *where* $\Pi =\;\text{diag}{\left(\pi \left(E\right)\right)}_{E\subseteq E}$.

***Proof:*** Note that, up to the scaling factor $\prod_{e\notin T}\sqrt{p_{e}\left(1-p_{e}\right)}$ which is uniform in E⊆E, it holds $\psi_{T}=\phi_{T}\Pi^{-1/2}$. Thus $\psi_{T}Q=\phi_{T}P\Pi^{-1/2}=\lambda_{T}\psi_{T}$, and it follows that ${\left\{\psi_{T}\right\}}_{T\subseteq E}$ forms a complete collection of eigenvectors of Q.

The symmetry of Q guarantees orthogonality of $\psi_{T},\psi_{T^{\prime }}$ provided $\lambda_{T}\neq \lambda_{T^{\prime }}$; however the spectrum of P will in general contain many repeated eigenvalues (*Remark* 4.2) and thus we check orthogonality in general as follows. For a given subset E⊆E, let $1_{E}\in R^{E}$ denote the indicator vector of *E*. Then for any T⊆E fixed, the vector *ϕ*_*T*_ may be recast as a product over edges in the form

$$\begin{array}{c} \phi_{T}\left(E\right)=\prod_{e\in E}g_{e,T}\left(1_{E}\left(e\right)\right),\;E\subseteq E, \end{array}$$

where $g_{e,T}:\left\{0,1\right\}\to R$ is given by

$$\begin{array}{c} g_{e,T}\left(x\right)=\left\{\begin{array}{cc} p_{e}, & x=1\;\text{and}\;e\in T,\\ 1-p_{e}, & x=0\;\text{and}\;e\in T,\\ 1, & x=1\;\text{and}\;e\notin T,\\ -1, & x=0\;\text{and}\;e\notin T. \end{array}\right.. \end{array}$$

Hence, for S,T⊆E fixed, it holds

$$\begin{array}{cc} \left\langle \psi_{T},\psi_{S}\right\rangle & =\sum_{E\subseteq E}\psi_{T}\left(E\right)\psi_{S}\left(E\right)\\ & =\prod_{e\notin T}\sqrt{p_{e}\left(1-p_{e}\right)}\;\prod_{e\notin S}\sqrt{p_{e}\left(1-p_{e}\right)}\\ & \times \sum_{X\in {\left\{0,1\right\}}^{E}}\frac{\prod_{e}g_{e,T}\left(X\left(e\right)\right)\;g_{e,S}\left(X\left(e\right)\right)}{\pi (E)}, \end{array}$$

where we identify subsets with their indicator vectors. Focusing on the last term for a moment, we have the factorization:

$$\begin{array}{cc} \sum_{X\in {\left\{0,1\right\}}^{E}} & \frac{\prod_{e\in E}g_{e,T}\left(X\left(e\right)\right)\;g_{e,S}\left(X\left(e\right)\right)}{\pi (E)}\\ & =\sum_{X\in {\left\{0,1\right\}}^{E}}\prod_{e\in E}\frac{g_{e,T}\left(X\left(e\right)\right)g_{e,S}\left(X\left(e\right)\right)}{p_{e}^{X(e)}{\left(1-p_{e}\right)}^{1-X(e)}}\\ & =\prod_{e\in E}(\sum_{x=0}^{1}\frac{g_{e,T}\left(x\right)\;g_{e,S}\left(x\right)}{p_{e}^{x}{\left(1-p_{e}\right)}^{1-x}}). \end{array}$$

This can be seen by expanding $\sum_{X\in {\left\{0,1\right\}}^{E}}\left(\cdot \right)$ into |E| nested sums and then rearranging. If *T* ≠ *S*, choose an edge $e^{*}$ on which they differ; assuming without loss of generality that $e^{*}\in T$ and $e^{*}\notin S$, one has

$$\begin{array}{cc} \sum_{x=0}^{1}\frac{g_{e^{*},T}\left(x\right)\;g_{e^{*},S}\left(x\right)}{p_{e^{*}}^{x}{\left(1-p_{e^{*}}\right)}^{1-x}} & =-\frac{1-p_{e^{*}}}{1-p_{e^{*}}}+\frac{p_{e^{*}}}{p_{e^{*}}}=0. \end{array}$$

so $\langle \psi_{T},\psi_{S}\rangle =0$. On the other hand, if *T* = *S*, then for each *e* one checks

$$\begin{array}{cc} \sum_{x=0}^{1}\frac{g_{e,T}{\left(x\right)}^{2}}{p_{e}^{x}{\left(1-p_{e}\right)}^{1-x}} & =\left\{\begin{array}{cc} \frac{{\left(1-p_{e}\right)}^{2}}{1-p_{e}}+\frac{p_{e}^{2}}{p_{e}}=1, & e\in T,\\ \frac{1}{1-p_{e}}+\frac{1}{p_{e}}=\frac{1}{p_{e}\left(1-p_{e}\right)}, & e\notin T. \end{array}\right. \end{array}$$

Therefore

$$\begin{array}{cc} \left\langle \psi_{T},\psi_{T}\right\rangle & =\prod_{e\notin T}p_{e}\left(1-p_{e}\right)\prod_{e\notin T}\frac{1}{p_{e}\left(1-p_{e}\right)}=1, \end{array}$$

which completes the proof.

Next we present an application to spectral representations of commute times for the simple edit process with edge probabilities $p={\left(p_{e}\right)}_{e\in E}$. To this end, consider a finite state space X={1,⋯,N} and a Markov chain ${\left(X_{t}\right)}_{t\geq 0}$ on X. For x∈X, we define the (possibly infinite) hitting time

$$\begin{array}{cc} \tau_{x} & =\;\inf\{t\geq 0:X_{t}=x\}. \end{array}$$

The expected hitting times and expected commute times are given, respectively, by

$$\begin{array}{cc} H(x,y) & =E\left[\tau_{y}|X_{0}=x\right],\\ C(x,y) & =H(x,y)+H(y,x), \end{array}$$

for each x,y∈X. We remark that in the case of our evolving graph processes, the commute time can be considered a notion of distance between subgraphs of the host graph, and is related to the effective resistance distance on the state graph. For completeness we recall the following lemma, which is well known and stated without proof (we refer the reader to ref. 38, theorem 3.1 for an analogous result on graphs).

Lemma 6.3. *Let* X={1,⋯,N} *be a finite state space and let* *P* *be the transition probability matrix of an ergodic reversible Markov chain* ${\left(X_{t}\right)}_{t\geq 0}$ *on* X*, and let* *π* *be its stationary distribution. Set* Π=diag(π(1),⋯,π(N)) *and* $Q=\Pi^{1/2}P\Pi^{-1/2}$*.* *Assume* *Q* *admits the row vector eigendecomposition*

$$\begin{array}{c} Q=\Psi^{⊤}\Lambda \Psi ,\;\Psi =\left[\begin{array}{c} \psi_{1}\\ \psi_{2}\\ \vdots \\ \psi_{N} \end{array}\right],\;\psi_{1}=\Pi^{1/2}1, \end{array}$$

*where* $1=\lambda_{1}>\lambda_{2}\geq \cdots \geq \lambda_{N}>-1$*,* *and define the normalized vectors* $\varphi_{k}=\Pi^{-1/2}\psi_{k}$ *so* $\varphi_{k}\left(i\right)=\psi_{k}\left(i\right)/\sqrt{\pi (i)}$*.* *Then for* x,y∈X*,* *fixed, the hitting time* H(x,y) *admits the following spectral representation:*

[20] $$\begin{array}{cc} H(x,y) & =\sum_{k=2}^{N}\frac{1}{1-\lambda_{k}}\varphi_{k}\left(y\right)\left(\varphi_{k}\left(y\right)-\varphi_{k}\left(x\right)\right). \end{array}$$

*Similarly, the commute time admits the spectral representation*

[21] $$\begin{array}{cc} C(x,y) & =\sum_{k=2}^{N}\frac{1}{1-\lambda_{k}}{\left(\varphi_{k}\left(x\right)-\varphi_{k}\left(y\right)\right)}^{2}. \end{array}$$

The following theorem gives a closed-form spectral representation of the commute time between subgraphs in the setting of the graph edit Markov chain.

Theorem 6.4. *Let* H=(V,E) *be a fixed host graph and let* $p={\left(p_{e}\right)}_{e\in E}$ *be fixed with* $0<p_{e}<1$ *for each* e∈E*.* *Let* ${\left(G_{t}\right)}_{t\geq 0}$ *be obtained from the simple edit process with edge probabilities* $p={\left(p_{e}\right)}_{e\in E}$*,* *let* *π* *denote the corresponding stationary distribution, and let* ${\left\{\psi_{T}\right\}}_{T\subseteq E}$ *denote the eigenvectors of the symmetrized transition probability matrix as in* *Theorem 6.2.* *Letting* E,F⊆E *be fixed, the commute time* C(E,F) *admits the following spectral representation:*

[22] $$\begin{array}{c} C\left(E,F\right)=\sum_{\begin{array}{c} T\cap (E▵F)\neq \emptyset \\ T\neq E \end{array}}\frac{m}{m-|T|}{\left(\frac{\psi_{T}\left(E\right)}{\sqrt{\pi (E)}}-\frac{\psi_{T}\left(F\right)}{\sqrt{\pi (F)}}\right)}^{2}, \end{array}$$

*where* E▵F *is the symmetric difference of* *E, F.*

***Proof:*** By *Theorem* 6.2 and *Lemma* 6.3, it follows that

[23] $$\begin{array}{c} C\left(E,F\right)=\sum_{\begin{array}{c} T\subseteq E\\ T\neq E \end{array}}\frac{m}{m-|T|}{\left(\frac{\psi_{T}\left(E\right)}{\sqrt{\pi (E)}}-\frac{\psi_{T}\left(F\right)}{\sqrt{\pi (F)}}\right)}^{2}. \end{array}$$

It remains to show that the sum in Eq. **23** can be taken over only the subsets that have nontrivial intersection with E▵F. It is enough to show that if T∩(E▵F)=∅, then

[24] $$\begin{array}{c} \frac{\phi_{T}\left(E\right)}{\pi (E)}=\frac{\phi_{T}\left(F\right)}{\pi (F)}, \end{array}$$

where *ϕ*_*T*_ is as in *Theorem* 6.1. We compute, again using *Theorem* 6.1,

$$\begin{array}{cc} \frac{\phi_{T}\left(E\right)}{\pi (E)} & =\frac{{\left(-1\right)}^{\left|E\setminus (E\cup T)\right|}\prod_{e\in T\cap E}p_{e}\prod_{e\in T\setminus E}\left(1-p_{e}\right)}{\prod_{e\in E}p_{e}\prod_{e\notin E}\left(1-p_{e}\right)} \end{array}$$

[25] $$\begin{array}{cc} & =\prod_{e\in E\setminus T}p_{e}^{-1}\prod_{e\notin (E\cup T)}{\left(p_{e}-1\right)}^{-1}\\ & =\prod_{e\notin T}\left(p_{e}^{-1}1_{\left\{e\in E\right\}}+{\left(p_{e}-1\right)}^{-1}1_{\left\{e\notin E\right\}}\right) \end{array}$$

and similarly for $\frac{\phi_{T}\left(F\right)}{\pi (F)}$. Now suppose *e* ∉ *T* is fixed such that e∉E▵F. Then e∈E∩F or e∉E∪F. In the former case,

$$\begin{array}{cc} & p_{e}^{-1}1_{\left\{e\in E\right\}}+{\left(p_{e}-1\right)}^{-1}1_{\left\{e\notin E\right\}}\\ & =p_{e}^{-1}1_{\left\{e\in F\right\}}+{\left(p_{e}-1\right)}^{-1}1_{\left\{e\notin F\right\}}=p_{e}^{-1}, \end{array}$$

and similarly in the latter case. Therefore, if $T\subseteq {\left(E▵F\right)}^{c}$ each term in the product which appears in Eq. **25** coincides and Eq. **24** holds, so that Eq. **22** is proved.

We now describe a small example which shows *Theorems* 6.1 and 6.4 in action.

**Fig. 5.**
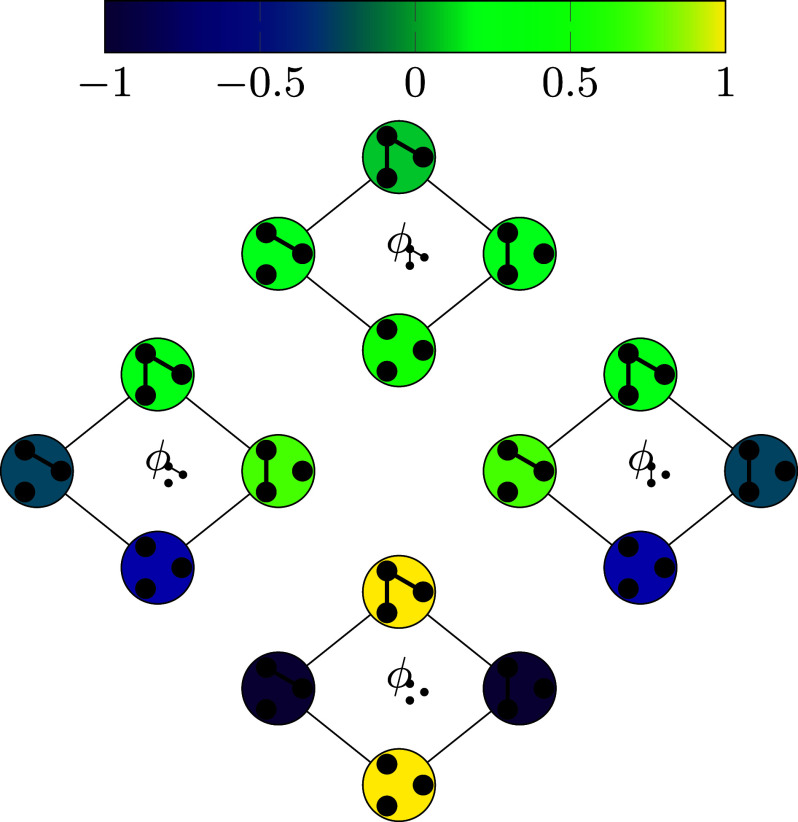
A drawing of the state graph and the corresponding eigenvectors *ϕ*_*T*_ of the transition probability matrix for the simple edit process with edge probabilities $p={\left(p_{e}\right)}_{e\in E}$ on a host graph H consisting of a path with two edges. We use *p* = 0.25 for concreteness. The eigenvectors *ϕ*_*T*_ are obtained using the formula in *Theorem* 6.1, and each copy of the state graph S(H) is indexed by a choice of *T*, rendered visually as a subscript of the symbol *ϕ* in each copy.

Example 6.5: In this example, we obtain the eigenvectors and commute time matrix of the transition probability matrix P associated to a simple edit process on a small host graph. Let H=({1,2,3},{{1,2},{2,3}}) consist of a path graph on three nodes and two edges, illustrated below. We symbolically denote the two edges *a*, *b*, respectively, for brevity in the discussion to follow.

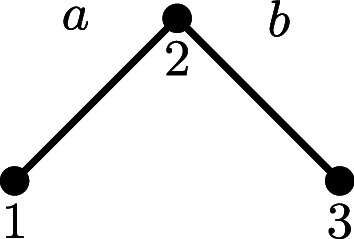

The edit semigroup is generated by the set $\left\{a^{+},a^{-},b^{+},b^{-}\right\}$, the elements of which are organized in the table below based on their reduced edit enumerations.

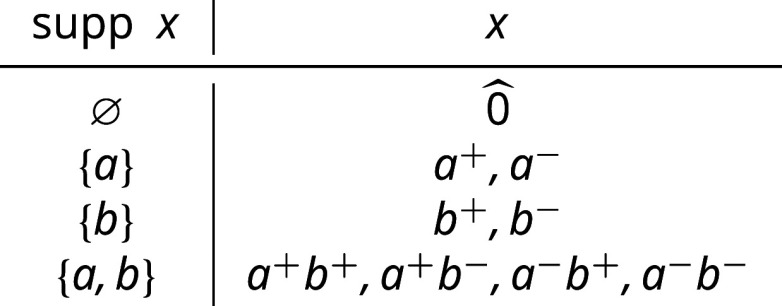

The corresponding lattice is the Boolean algebra on $2^{\left\{a,b\right\}}$, which has exactly four elements (themselves shown in the leftmost column of the table above). From *Lemma* 3.6, we know that the chambers C of S are exactly the elements which have support of maximal cardinality, which in this case is two. Fixing $p_{a},p_{b}\in \left(0,1\right)$, the transition probability matrix P for the simple edit process (equivalently, the random walk on the chambers of S) is given by

$$\begin{array}{c} P=\left[\begin{array}{cccc} \frac{p_{a}+p_{b}}{2} & \frac{1-p_{b}}{2} & \frac{1-p_{a}}{2} & 0\\ \frac{p_{b}}{2} & \frac{1+p_{a}-p_{b}}{2} & 0 & \frac{1-p_{a}}{2}\\ \frac{p_{a}}{2} & 0 & \frac{1-p_{a}+p_{b}}{2} & \frac{1-p_{b}}{2}\\ 0 & \frac{p_{a}}{2} & \frac{p_{b}}{2} & 1-\frac{p_{a}+p_{b}}{2} \end{array}\right], \end{array}$$

where the rows and columns are indexed by $\left\{a^{+}b^{+},a^{+}b^{-},a^{-}b^{+},a^{-}b^{-}\right\}$. By *Lemma* 2.1, we know that the stationary distribution *π* is given by

$$\begin{array}{c} \pi =\left[\begin{array}{cccc} p_{a}p_{b} & p_{a}\left(1-p_{b}\right) & \left(1-p_{a}\right)p_{b} & \left(1-p_{a}\right)\left(1-p_{b}\right) \end{array}\right]. \end{array}$$

and that $\lambda_{\left\{a,b\right\}}=1$. From *Theorem* 6.1, we have

$$\begin{array}{cc} \phi_{\left\{b\right\}} & =\left[\begin{array}{cccc} p_{b} & \left(1-p_{b}\right) & -p_{b} & -(1-p_{b}) \end{array}\right],\;\lambda_{\left\{b\right\}}=\frac{1}{2}, \end{array}$$

$$\begin{array}{cc} \phi_{\left\{a\right\}} & =\left[\begin{array}{cccc} p_{a} & -p_{a} & \left(1-p_{a}\right) & -(1-p_{a}) \end{array}\right],\;\lambda_{\left\{a\right\}}=\frac{1}{2},\\ \phi_{\emptyset } & =\left[\begin{array}{cccc} 1 & -1 & -1 & 1 \end{array}\right],\;\lambda_{\emptyset }=0. \end{array}$$

Thus we have the diagonalization $P=U^{-1}\Lambda U$ of P, where

[26] $$\begin{array}{c} U=\left[\begin{array}{cccc} p_{a}p_{b} & p_{a}\left(1-p_{b}\right) & \left(1-p_{a}\right)p_{b} & \left(1-p_{a}\right)\left(1-p_{b}\right)\\ p_{b} & \left(1-p_{b}\right) & -p_{b} & -(1-p_{b})\\ p_{a} & -p_{a} & \left(1-p_{a}\right) & -(1-p_{a})\\ 1 & -1 & -1 & 1 \end{array}\right]. \end{array}$$

and Λ is the diagonal matrix of eigenvalues given by

[27] $$\text{Λ}=\left[\begin{array}{llll} 1 & & & \\ & \frac{1}{2} & & \\ & & \frac{1}{2} & \\ & & & 0 \end{array}\right].$$

Finally, by applying *Theorem* 6.4 together with the eigenvalues and eigenvectors from Eqs. **26** and **27**, the matrix of commute times $C\in R^{4\times 4}$ with entries C(E,F) for each E,F⊆E can be computed. For simplicity we include the matrix *C* in the case $p_{a}=p_{b}=p$ below.

$$\begin{array}{c} C=\left[\begin{array}{cccc} 0 & \frac{1+p}{p^{2}\left(1-p\right)} & \frac{1+p}{p^{2}\left(1-p\right)} & \frac{1}{p^{2}{\left(1-p\right)}^{2}}\\ \frac{1+p}{p^{2}\left(1-p\right)} & 0 & \frac{4}{p(1-p)} & \frac{2-p}{p{\left(1-p\right)}^{2}}\\ \frac{1+p}{p^{2}\left(1-p\right)} & \frac{4}{p(1-p)} & 0 & \frac{2-p}{p{\left(1-p\right)}^{2}}\\ \frac{1}{p^{2}{\left(1-p\right)}^{2}} & \frac{2-p}{p{\left(1-p\right)}^{2}} & \frac{2-p}{p{\left(1-p\right)}^{2}} & 0 \end{array}\right]. \end{array}$$

## 7. Discussion

In this paper, we have investigated several edit-based graph evolving processes which satisfy the memoryless conditions. By using methods from semigroup spectral theory, we give a detailed spectral analysis of the transition probability matrices associated to both simple and compound edit processes. We remark that it is conceivable that other examples of memoryless processes could be developed or identified in other areas of interest, for example statistical physics, where sharp mixing rate analyses of sampling algorithms are of interest (39, 40). Further questions can be asked about extensions and modifications, as well as comparisons of memoryless processes with others.

## Data Availability

All study data are included in the main text.
